# Exploring the intersection of functional recurrence, patient-reported sexual function, and treatment satisfaction after anterior buccal mucosal graft urethroplasty

**DOI:** 10.1007/s00345-021-03648-y

**Published:** 2021-03-11

**Authors:** Malte W. Vetterlein, Almut Gödde, Valentin Zumstein, Philipp Gild, Phillip Marks, Armin Soave, Christian P. Meyer, Silke Riechardt, Roland Dahlem, Margit Fisch, Luis A. Kluth, for the Trauma and Reconstructive Urology Working Party of the European Association of Urology Young Academic Urologists

**Affiliations:** 1 Department of Urology, University Medical Center Hamburg-Eppendorf, Martinistr. 52, 20246 Hamburg, Germany; 2 Department of Urology, School of Medicine, University of St, Gallen, St. Gallen, Switzerland; 3 Department of Urology, University Medical Center, Frankfurt, Frankfurt (Main), Germany

**Keywords:** Patient Reported Outcome Measures, Patient Satisfaction, Recurrence, Sexual Dysfunction, Urethral Stricture

## Abstract

**Purpose:**

To evaluate the interplay of stricture recurrence, sexual function, and treatment satisfaction after substitution urethroplasty.

**Methods:**

Observational study of men undergoing 1-stage buccal mucosal graft urethroplasty for anterior urethral stricture between 2009 and 2016. Patients were dichotomized by self-reported treatment satisfaction. Sexual function was assessed by validated and non-validated patient-reported outcome measures. Functional recurrence was defined as symptomatic need of re-intervention. Bivariate analyses, Kaplan–Meier estimates, qualitative and quantitative analyses by uni- and multivariable regression were employed to evaluate the interplay of sexual function, functional recurrence, and treatment satisfaction.

**Results:**

Of 534 men with bulbar (82%), penobulbar (11%), and penile strictures (7.3%), 451 (84%) were satisfied with the surgery. There were no differences in stricture location, previous treatment, graft length, or surgical technique between satisfied and unsatisfied patients (all *p * ≥  0.2). Recurrence-free survival was 85% at a median follow-up of 33 mo and decreased significantly with each Likert item towards increasing dissatisfaction (*p * <  0.001). Dissatisfied patients more often reported postoperative loss of rigidity, tumescence, reduced ejaculatory volume, ejaculatory pain, and reduced penile length (all *p * ≤  0.042). In 83 dissatisfied men, functional recurrence (28%) and oral morbidity (20%) were the main drivers of dissatisfaction in qualitative analysis. Multivariable analyses revealed functional recurrence and impaired postoperative ejaculatory function as independent predictors of treatment dissatisfaction (all *p * ≤  0.029) after adjusting for confounders.

**Conclusion:**

We found an association of both functional success and sexual function with patient-reported treatment satisfaction after substitution urethroplasty. Such findings validate the clinical significance of defining the symptomatic need for re-intervention as an endpoint and underline the importance of further research evaluating sexual function before and after open urethral reconstruction.

**Supplementary Information:**

The online version contains supplementary material available at 10.1007/s00345-021-03648-y.

## Introduction

There are differences in the perception of successful surgery between patients who undergo urethroplasty and surgeons who perform the procedure [1]. The importance of patient-reported outcome measures (PROMs) has been scrutinized over the last decade [2, 3], culminating in the development of the Urethral Stricture Symptom and Impact Measure (USSIM) to incorporate both surgeons’ and patients’ perspective on important outcomes after urethroplasty [4]. Whereas the significance of generic quality of life tools for patients undergoing urethroplasty is controversial [2, 5], treatment satisfaction is more or less a universally accepted surrogate of patient-centered success and has been incorporated in several recent urethroplasty outcome analyses [1, 2, 6, 7]. Patient satisfaction rates after urethroplasty are fairly high and range between 78 and 87% [1, 2, 6, 7]. However, the driving forces of dissatisfaction with the surgical outcome have not unanimously been identified and there are several potential reasons for the heterogeneity of the available evidence such as the variety of surgical techniques used or the definition of objective surgical success [1, 6, 7]. Experts agree on the crucial value of evaluating sexual function after urethroplasty as it has been shown that penile anatomy, erectile, and ejaculatory function may be altered by urethroplasty [8, 9] through the dissection of bulbospongiosus and ischiocavernosus muscles and—most importantly—may affect patient-reported treatment satisfaction postoperatively [1, 6, 7, 10].

Against this backdrop, our study aim was threefold: First, we aimed to leverage a contemporary, homogeneous series of patients undergoing 1-stage buccal mucosal graft urethroplasty (BMGU) to assess both objective and subjective treatment outcomes. Second, we focused on functional recurrence, sexual function, and treatment satisfaction evaluating the interplay of those factors. Additionally we focused on the identification of the underlying reasons of patient-reported dissatisfaction. Third, we aimed to assess whether outcomes differed between different stricture locations. We hypothesized that functional recurrence and postoperative self-reported sexual function would significantly impact treatment satisfaction and that there would be differences after stratifying patients by stricture location in the anterior urethra.

## Patients and methods

### Study population

Overall, 1039 BMGU cases were captured in our retrospective database between 2009 and 2016 after institutional review board approval and patient consent. We excluded patients with posterior, meatal and distal strictures, or those with multiple stricture locations, as well as patients with a history of radiotherapy, gender reassignment surgery, hypospadias, and lichen sclerosus. The selection process is depicted in Supplementary Fig. S1. Our final study population consisted of 534 men undergoing 1-stage BMGU for anterior urethral stricture.

### Evaluation, surgical procedures, and perioperative management

Preoperatively, all patients were evaluated via medical history, physical examination, urine culture, uroflowmetry, post-void residual urine volume, and combined retrograde urethrography and voiding cystourethrography [11–13]. The choice of the technique used was at the surgeon’s discretion based on stricture location, length, history of previous treatments, and intraoperative findings. According to our institutional standard, ventral onlay BMGU [14] is performed for bulbar stricture repair and for most penobulbar strictures with a significant proportion of bulbar involvement. Penile strictures are commonly approached by inlay procedures as described by Asopa et al. [15, 16] or our modification [17] of the dorsal inlay urethroplasty as described by Barbagli et al. [18] In all patients, a standardized institutional perioperative pathway was applied [12, 13]. We performed suprapubic (21 days) plus transurethral catheterization (10 days) or transurethral catheterization only (21 days) in patients without previous urethroplasty and for redo urethroplasties, respectively. Transurethral micturition started if urethral integrity without evidence of extravasation could be demonstrated at urethrography 21 days postoperatively [12]. In case of extravasation, the catheter remained for another two weeks until repeat urethrography.

### Follow-up, sexual function, treatment satisfaction, and stricture recurrence

Cross-sectional postoperative follow-up by mail and phone was performed using parts of the psychometrically validated and extended German translation [19] of a previously developed and validated urethra stricture-specific PROM instrument [2]. We specifically focused on patient-reported outcomes regarding sexual function and treatment satisfaction.

Select questions evaluating sexual function were adapted from previous studies, using validated [20, 21] and non-validated questions [22] regarding sexual function (i.e., erection and ejaculation [20], penile length and deviation) [21], and perineal neuropathy or numbness [22]. Sexual dysfunction was defined as any self-reported deterioration of above mentioned sexual function parameters. Further, patients were asked if sexual dysfunction impacts their daily life.

A global treatment satisfaction question assessed whether patients were satisfied with the surgical outcome with answers on a 5-item Likert scale. The items were dichotomized as previously reported: (1) satisfied: patients reporting being “very satisfied” and “satisfied” vs. (2) unsatisfied: patients reporting “neither satisfied or unsatisfied”, “unsatisfied”, or “very unsatisfied” [7]. The underlying reasons of dissatisfaction were captured using an open-ended format style of questioning as previously described [10].

Functional stricture recurrence (i.e., symptomatic need of any postoperative instrumentation during follow-up, including dilation, endoscopic or reconstructive surgery) [12, 13, 23] was assessed by both medical chart review and patient interrogation.

### Covariables

Patient baseline characteristics, such as age at surgery, American Society of Anesthesiologists (ASA™) physical status, body mass index, and comorbidities were abstracted. We, furthermore, assessed surgical characteristics, such as stricture location (bulbar vs. penobulbar vs. penile), previous treatment (none vs. any direct vision internal urethrotomy vs. urethroplasty ± urethrotomy), length of the buccal mucosal graft, and the surgical technique used (onlay vs. inlay).

## Statistical analyses

First, baseline and surgical characteristics were stratified by treatment satisfaction. Means and standard deviations (SDs) were reported for continuous variables and frequencies and proportions for categorical variables.

Second, reverse Kaplan–Meier estimates were calculated for follow-up time in censored patients. Kaplan–Meier curves were plotted to depict recurrence-free survival in the overall cohort and stratified by treatment satisfaction as well as stricture location. Equality of the curves was tested by the log-rank test.

Third, we reported frequencies and proportions of sexual function parameters stratified according to treatment satisfaction and stricture location, and outcomes were compared across the different groups.

Finally, we calculated crude odds ratios (ORs) using univariable logistic regression analyses to estimate the association between covariables and sexual function parameters with treatment dissatisfaction. To identify independent predictors of treatment dissatisfaction, we finally employed a multivariable logistic regression model.

For all descriptive analyses, differences between groups were evaluated using the* t*-test, analysis of variance (ANOVA), Pearson’s χ^2^ test, or Fisher’s exact test when samples were < 10, as appropriate.

All analyses were performed using Stata® (StataCorp. 2015. Stata Statistical Software: Release 14. College Station, TX: StataCorp LP). Two-sided statistical significance was defined as a *p* value < 0.05.

## Results

### Baseline and surgical characteristics

Mean patient age was 52 ± 17 yr and a total of 434 patients (81%) had undergone previous treatment (previous urethroplasty in 18%). Mean graft length was 4.8 ± 1.8 cm, and 493 (92%) vs. 41 patients (7.7%) were operated using an onlay vs. inlay technique, respectively.

Stratification of stricture location revealed 438 bulbar (82%), 57 penobulbar (11%), and 39 penile strictures (7.3%). Overall, 451 patients (84%) were satisfied with the surgical outcome. There was a shift towards a higher comorbidity burden (age, hypertension, and diabetes) in patients reporting dissatisfaction compared to their satisfied counterparts (all *p* ≤ 0.026; Supplementary Table S1). There were no differences in surgical characteristics between satisfied and unsatisfied patients (all *p* ≥  0.2; Supplementary Table S1).

### Recurrence-free survival analyses

**Fig. 1:**
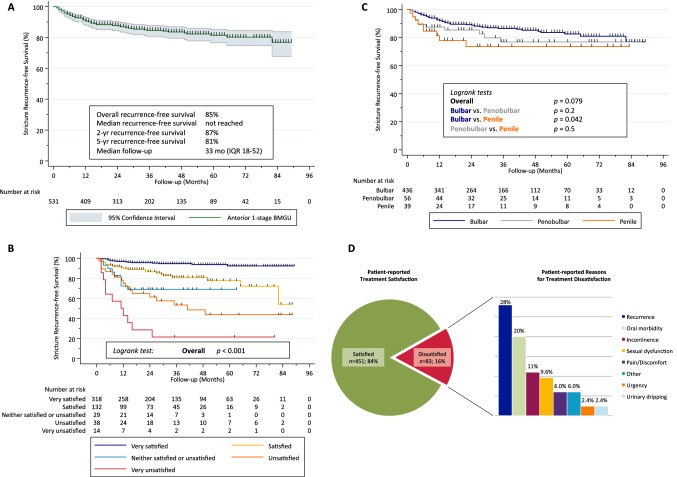
Kaplan–Meier estimates of stricture recurrence-free survival in men who underwent anterior 1-stage buccal mucosal graft urethroplasty **a** in the overall cohort, **b** stratified by postoperative patient-reported treatment satisfaction, and **c** stratified by stricture location. **d** Patient-reported treatment satisfaction and reasons for dissatisfaction with the surgical outcome in 83 of 534 men undergoing 1-stage buccal mucosal graft urethroplasty for anterior urethral stricture. (Pain refers to perineal pain/discomfort in the surgical field)

Overall recurrence-free survival was 85% (*n* = 454) at a median follow-up of 33 mo (Fig. 1a). This translated into 2-yr and 5-yr recurrence-free survival of 87% and 81%, respectively. Recurrence-free survival decreased significantly with each Likert item towards increasing patient-reported dissatisfaction (*p* < 0.001; Fig. 1b). For bulbar, penobulbar, and penile strictures overall recurrence-free survival was 87%, 79%, and 77%, respectively. This translated into 2-yr and 5-yr recurrence-free survival of 89% and 83% for bulbar, 85% and 77% for penobulbar, and 74% and 74% for penile strictures, respectively (Fig. 1c). Recurrence-free survival was significantly shorter in penile vs. bulbar strictures (*p* = 0.042), whereas there was no difference between bulbar and penobulbar (*p* = 0.2) or penobulbar and penile strictures (*p* = 0.5; Fig. 1c).

### Postoperative sexual function

Median time of patient-reported functional outcomes assessment was 29 mo postoperatively (IQR 13–48). At the postoperative assessment, 65% of patients reported a sufficient erection, 92% had an adequate glanular tumescence, 70% reported no change in ejaculatory volume, and 92% did not experience any ejaculatory pain. Likewise, the majority of patients did not experience scrotal or perineal numbness (91%), and did not recognize a change in erection angle (92%) or penile length (90%). In 75% of patients, sexual symptoms did not at all interfere with their everyday life, whereas 12%, 7.4%, and 6.3% reported a little, moderate, and extreme interference, respectively (Supplementary Table S2). If patients reported a decreased erectile or ejaculatory function postoperatively, we asked them if those symptoms had worsened over time compared to the preoperative status. Of 85 (erectile dysfunction) and 43 (ejaculatory dysfunction) patients with available data, 31% and 9.3% referred to a postoperative deterioration of rigidity and ejaculatory volume, respectively (Supplementary Table S2).

There were distinct differences regarding sexual function between satisfied and unsatisfied patients. Particularly, satisfied patients significantly more often reported sufficient rigidity and tumescence, unchanged ejaculatory volume, absence of ejaculatory pain, unchanged penile length, and less interference of sexual symptoms with their everyday life compared to patients who were dissatisfied with the surgical outcome (all *p * ≤  0.042; Supplementary Table S2).

For most of the sexual function parameters there was no difference in the distributions of answers between the different stricture locations. However, patients with penile strictures significantly more often claimed a change in penile angle (28% vs. 7.4% vs. 6.0%; *p* < 0.001) and penile length (26% vs. 7.7% vs. 9.1%; *p* = 0.011) compared to penobulbar and bulbar strictures, respectively. Similarly, patients with penile strictures more often reported an impaired erectile function (reduced rigidity or no erection) compared to those with penobulbar and bulbar strictures (48% vs. 39% vs. 33%; *p* = 0.006; Supplementary Table S3).

### Treatment satisfaction

Qualitative analyses in 83 dissatisfied patients revealed functional stricture recurrence (28%) and oral morbidity (20%) as the main postoperative drivers of dissatisfaction with the surgical outcome (Fig. 1d). The multifaceted characteristics of oral morbidity in dissatisfied patients are presented in Supplementary Table S4.

In univariable logistic regression analyses, increasing age, functional stricture recurrence, postoperative change in penile length, and both impaired postoperative erectile and ejaculatory function were associated with treatment dissatisfaction (all *p * ≤  0.045). After adjusting for other potentially confounding covariables, stricture recurrence and impaired postoperative ejaculatory function held true as independent predictors of patient-reported treatment dissatisfaction (all *p * ≤  0.029). Conversely, stricture location did not affect treatment dissatisfaction after multivariable adjustment (all *p * ≥  0.4). Further details on the regression models are depicted in Table 1.

**Table 1:** Logistic regression analyses predicting patient-reported treatment satisfaction after anterior one-stage buccal mucosal graft urethroplasty

Covariables	Univariable		Multivariable	
	Crude OR (95% CI)	*p* value	OR (95% CI)	*p* value
Age (years, continuous)	1.03 (1.02–1.05)	< 0.001	1.01 (0.98–1.03)	0.7
Previous treatment (Ref.: None)				
DVIU	0.83 (0.45–1.52)	0.5	1.00 (0.43–2.32)	> 0.9
Urethroplasty ± DVIU	1.04 (0.50–2.17)	> 0.9	0.55 (0.18–1.68)	0.3
Graft length (cm, continuous)	0.96 (0.83–1.10)	0.5	0.93 (0.76–1.13)	0.4
Stricture location (Ref.: Bulbar)				
Penobulbar	1.30 (0.64–2.64)	0.5	0.88 (0.26–3.03)	0.8
Penile	0.62 (0.21–1.81)	0.4	0.51 (0.13–1.92)	0.3
Stricture recurrence (Ref.: None)	8.86 (5.18–15.17)	< 0.001	9.41 (4.67–18.96)	< 0.001
Change in penile length (Ref.: None)	2.01 (1.02–3.96)	0.045	1.49 (0.52–4.23)	0.5
Change in penile angle (Ref.: None)	1.41 (0.63–3.19)	0.4	1.47 (0.46–4.63)	0.5
Impaired postoperative erectile function (Ref.: Sufficient rigidity)	3.08 (1.91–4.98)	< 0.001	0.93 (0.38–2.27)	0.9
Impaired postoperative ejaculatory function (Ref.: Unchanged ejaculatory volume)	3.38 (1.90–6.01)	< 0.001	2.35 (1.09–5.07)	0.029

*CI* confidence interval, *DVIU* direct vision internal urethrotomy, *OR* odds ratio

### Discussion

In more than 500 men undergoing 1-stage BMGU we confirmed high levels of treatment satisfaction, which precisely mirrored the objective functional success rate of 85%. Furthermore, we performed a detailed analysis on postoperative sexual function and found that roughly one-third of patients reported an impaired postoperative erectile or ejaculatory function. Both subjective sexual function parameters as well as objective functional treatment success were clearly associated with treatment satisfaction in multivariable analyses. Whereas such results corroborate the available evidence on treatment satisfaction and sexual function after urethroplasty at large, there are some findings, which deserve particular consideration.

It seems natural that patients will be more satisfied with the surgical outcome if they do not need a re-intervention. Interestingly, to the best of our knowledge, such association has not been clearly delineated in the literature to date. We did not only find functional recurrence to be highly predictive for treatment satisfaction as a binary variable in multivariable analysis, we further validated the discriminative ability of the subjective 5-item Likert scale on treatment satisfaction to mirror objective functional recurrence as shown in Fig. 1b. This finding has clinical implications. First, we corroborate the need and meaningfulness to incorporate patient-reported treatment satisfaction into every urethroplasty outcome analysis. Second, our findings strengthen the clinically obvious importance and scientific validity to define functional success as the symptomatic need of a re-intervention [24], as this appears to represent a major driver of treatment dissatisfaction (Fig. 1b and d, Table 1). Admittedly, uroflowmetry parameters and cystoscopy may be clinically more accurate to assess anatomic recurrence, but the impact of impaired voiding parameters on treatment satisfaction [3] as well as the significance [25] and patient compliance regarding sequential follow-up cystoscopies [26] is a matter of debate.

After stratifying patients according to stricture location, we found distinct differences in self-reported sexual function. Particularly, roughly one out of four patients with penile strictures reported a change of penile angle or length, significantly more often than their counterparts with (peno)bulbar strictures. This implies a substantial adverse effect of penile stricture location on sexual outcomes and adds to the limited evidence available [9]. Rates of penile shortening after BMGU range between 3.8% [27] up to 55% [28] with a tendency towards more pronounced penile shortening the more distally the stricture is located. An effect of stricture location on sexual function may be further substantiated by a greater proportion of patients with penile compared to (peno)bulbar strictures reporting an impaired postoperative erectile rigidity. However, we acknowledge that those findings do not allow for unambiguously concluding higher erectile dysfunction rates for penile strictures.

Sexual function in general and predominantly ejaculatory function had a significant effect on treatment satisfaction after multivariable adjustment. Descriptive analyses also revealed certain differences regarding other sexual symptoms between satisfied and unsatisfied patients. Generally, data is scarce and heterogeneous on the course of a patient’s sexual function after undergoing urethroplasty, but experts agree on the multifactorial etiology with distinct differences depending on stricture length, location, and type of reconstruction [9]. Interestingly, men with diabetes or hypertension, which are acknowledged risk factors of erectile dysfunction, more frequently reported dissatisfaction in bivariate analyses. Such data further point to a multifaceted intersection of comorbidity, sexual function, and treatment satisfaction. Our findings underline the salient importance of assessing sexual function in young and middle-aged men undergoing urethroplasty, as there is a clear association with treatment satisfaction, which may be considered a surrogate of condition-related quality of life.

Our study is not devoid of limitations. First, given the cross-sectional, retrospective study design, we did not collect preoperative PROMs and thus, we were not able to evaluate longitudinal changes of patient-reported outcomes at predefined time points to clearly infer causal associations. Although we asked patients whether erectile and ejaculatory symptoms had changed following surgery, this approach might be prone to recall bias. Additionally, we did not assess whether patient-reported sexual dysfunction did occur before or after applying potential counter-measures, such as PDE5 inhibitors, traction or vacuum devices. Further, as we did not systematically assess the generic quality of life indicators such as the EQ visual analogue scale, there may be further multifactorial reasons to predict dissatisfaction, which we were not able to uncover.

Second, our institution is a tertiary referral center and thus, many patients are subsequently treated by a private practice urologist. A standardized follow-up assessment of objective parameters such as uroflowmetry and cystoscopy was not possible in the majority of patients. Hence, we were not able to directly compare the impact of objective (anatomic) vs. indirect/subjective (functional) success on treatment satisfaction.

Third, our cohort is limited to patients undergoing BMGU. While such homogeneity and focus on a particular technique is per se one of the major strengths of our study, we acknowledge that our findings are not necessarily transferrable to other techniques. In this context, we believe it should be mentioned that stricture location in our patient population may be more or less considered a surrogate of the surgical technique used, given that almost all bulbar and penobulbar strictures were treated with an onlay, whereas penile strictures were treated with an inlay graft (Supplementary Table S1).

Fourth, to date, there is no readily available procedure-specific validated tool to assess sexual function following urethroplasty. Thus, non-validated sexual health questions were adapted from a several preexisting urethroplasty landmark studies to assess the associations with treatment satisfaction.

## Conclusions

We found a significant association of both objective functional success and subjective sexual function with patient-reported treatment satisfaction in over 500 men undergoing 1-stage BMGU for anterior urethral stricture. Further, sexual function parameters appear to differ depending on stricture location. Those findings validate the clinical significance of defining the symptomatic need for re-intervention as a meaningful indirect endpoint alongside established objective parameters such as uroflowmetry or cystoscopy. Further, they underline the importance and need for further research evaluating the course of sexual function before and after open urethral reconstruction.

## Supplementary Information

Below is the link to the electronic supplementary material.

Supplementary file1 (PDF 51 KB)

Supplementary file2 (PDF 50 KB)

Supplementary file3 (PDF 77 KB)

Supplementary file4 (PDF 80 KB)

Supplementary file5 (PDF 44 KB)
