# Supplementary material for: Exploring the intersection of functional recurrence, patient-reported sexual function, and treatment satisfaction after anterior buccal mucosal graft urethroplasty
Source: World J Urol. 2021 Mar 11;39(9):3533–9. doi: 10.1007/s00345-021-03648-y (PMC8510905; doi:10.1007/s00345-021-03648-y)
Supplement: Supplementary file 1 — Supplementary file1 (PDF 51 KB) [file 345_2021_3648_MOESM1_ESM.pdf]

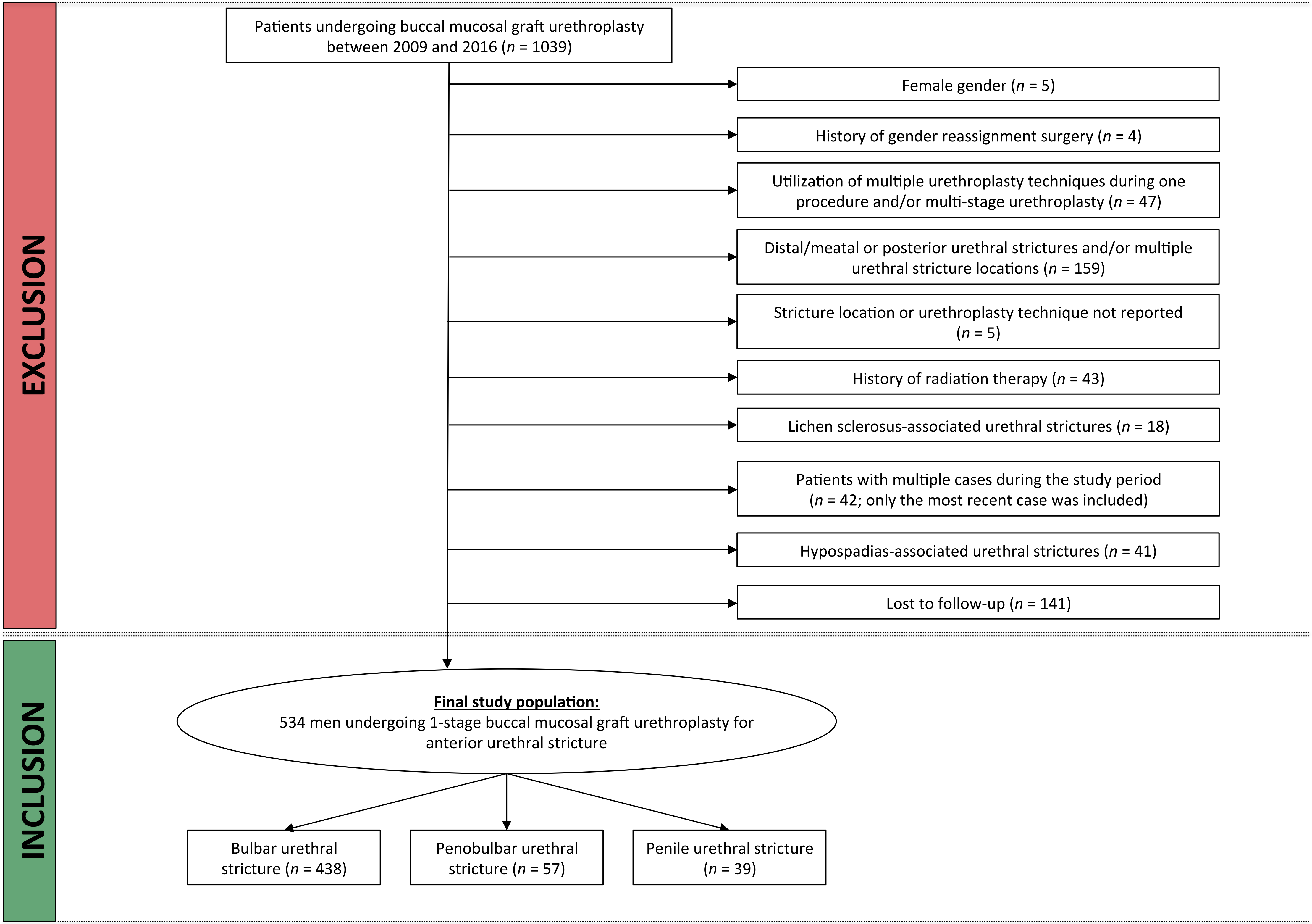
EXCLUSION
Patients undergoing buccal mucosal graft urethroplasty between 2009 and 2016 (n = 1039)
Female gender (n = 5)
History of gender reassignment surgery (n = 4)
Utilization of multiple urethroplasty techniques during one procedure and/or multi-stage urethroplasty (n = 47)
Distal/meatal or posterior urethral strictures and/or multiple urethral stricture locations (n = 159)
Stricture location or urethroplasty technique not reported (n = 5)
History of radiation therapy (n = 43)
Lichen sclerosus-associated urethral strictures (n = 18)
Patients with multiple cases during the study period (n = 42; only the most recent case was included)
Hypospadias-associated urethral strictures (n = 41)
Lost to follow-up (n = 141)
INCLUSION
Final study population:
534 men undergoing 1-stage buccal mucosal graft urethroplasty for anterior urethral stricture
Bulbar urethral stricture (n = 438)
Penobulbar urethral stricture (n = 57)
Penile urethral stricture (n = 39)
