# Supplementary material for: Exploring the intersection of functional recurrence, patient-reported sexual function, and treatment satisfaction after anterior buccal mucosal graft urethroplasty
Source: World J Urol. 2021 Mar 11;39(9):3533–9. doi: 10.1007/s00345-021-03648-y (PMC8510905; doi:10.1007/s00345-021-03648-y)
Supplement: Supplementary file 2 — Supplementary file2 (PDF 50 KB) [file 345_2021_3648_MOESM2_ESM.pdf]

Supplementary Table 1. Baseline and surgical characteristics of 534 men undergoing 1-stage buccal mucosal graft urethroplasty, stratified by treatment satisfaction.

	Overall (*n*=534)	Satisfied (*n*=451)	Unsatisfied (*n*=83)	*p* value
**Baseline characteristics**				
Age (yr); mean (SD)	52 (17)	51 (17)	59 (14)	<0.001
ASA™ physical status (*n*=525); *n* (%)				0.063
1-2	422 (80)	363 (82)	59 (73)	
3-4	103 (20)	81 (18)	22 (27)	
Body mass index (*n*=517); mean (SD)	27 (4.1)	27 (4.1)	27 (4.3)	0.6
Coronary heart disease (*n*=533); *n* (%)				0.7
No	481 (90)	407 (90)	74 (89)	
Yes	52 (9.8)	43 (9.6)	9 (11)	
Hypertension (*n*=533); *n* (%)				0.026
No	340 (64)	296 (66)	44 (53)	
Yes	193 (36)	154 (34)	39 (47)	
Diabetes mellitus (*n*=533); *n* (%)				0.014
No	482 (90)	413 (92)	69 (83)	
Yes	51 (9.6)	37 (8.2)	14 (17)	
**Surgical characteristics**				
Stricture location; *n* (%)				0.5
Bulbar	438 (82)	370 (82)	68 (82)	
Penobulbar	57 (11)	46 (10)	11 (13)	
Penile	39 (7.3)	35 (7.8)	4 (4.8)	
Previous treatment; *n* (%)				0.7
None	100 (19)	83 (18)	17 (20)	
DVIU	337 (63)	288 (64)	49 (59)	
Urethroplasty ± DVIU	97 (18)	80 (18)	17 (20)	
Length of graft in cm (*n*=529); mean (SD)	4.8 (1.8)	4.8 (1.8)	4.7 (1.6)	0.5
Surgical technique; *n* (%)				0.2
Onlay	493 (92)	413 (92)	80 (96)	
Inlay	41 (7.7)	38 (8.4)	3 (3.6)	

ASA™, American Society of Anesthesiologists; DVIU, direct vision internal urethrotomy; SD, standard deviation;
Proportions may not add up to 100%, as they are rounded.
