# Supplementary material for: Exploring the intersection of functional recurrence, patient-reported sexual function, and treatment satisfaction after anterior buccal mucosal graft urethroplasty
Source: World J Urol. 2021 Mar 11;39(9):3533–9. doi: 10.1007/s00345-021-03648-y (PMC8510905; doi:10.1007/s00345-021-03648-y)
Supplement: Supplementary file 3 — Supplementary file3 (PDF 77 KB) [file 345_2021_3648_MOESM3_ESM.pdf]

Supplementary Table 2. Patient-reported sexual function after anterior 1-stage buccal mucosal graft urethroplasty, stratified by treatment satisfaction.

**Sexual Function Parameters**; *n* (%)	Overall (*n*=534)	Satisfied (*n*=451)	Unsatisfied (*n*=83)	*p* value
In case you have been able to get an erection, how would you rate the hardness of your erection? (*n*=531; 99%)				<0.001
Sufficient rigidity	345 (65)	310 (69)	35 (42)	
Reduced rigidity	96 (18)	78 (17)	18 (22)	
No erection	90 (17)	60 (13)	30 (36)	
How was your postoperative erectile function compared to preoperatively, if rigidity was reduced or if there was no erection after surgery? (*n*=85; 46%)				>0.9
Unchanged	59 (69)	39 (70)	20 (69)	
Worsened	26 (31)	17 (30)	9 (31)	
Does your glans fully swell during erection? (*n*=437; 82%)				0.026
Yes	401 (92)	358 (93)	43 (83)	
No	36 (8.2)	27 (7.0)	9 (17)	
How would you rate the amount or volume of semen when you ejaculate? (*n*=444; 83%)				<0.001
As much as it always was	312 (70)	287 (74)	25 (45)	
Reduced volume	77 (17)	60 (15)	17 (31)	
No ejaculation	55 (12)	42 (11)	13 (24)	
How was your postoperative ejaculatory function compared to preoperatively, if semen volume was reduced or if there was no ejaculation after surgery? (*n*=43; 33%)				>0.9
Unchanged	39 (91)	31 (91)	8 (89)	
Worsened	4 (9.3)	3 (8.8)	1 (11)	
In the last month, have you experienced any physical pain or discomfort when you ejaculated? (*n*=438; 82%)				0.039
No	402 (92)	353 (93)	49 (84)	
Yes	36 (8.2)	27 (7.1)	9 (16)	
Have you experienced scrotal or perineal numbness over the last couple of months? (*n*=528; 99%)				0.069
No	479 (91)	409 (92)	70 (85)	
Yes	49 (9.3)	37 (8.3)	12 (15)	
Has the angle of your erection changed after surgery? (*n*=512; 96%)				0.4
No	472 (92)	401 (93)	71 (90)	
Yes	40 (7.8)	32 (7.4)	8 (10)	
Has the length of your penis changed since your surgery? (*n*=508; 95%)				0.042
No	456 (90)	391 (91)	65 (83)	
Yes	52 (10)	39 (9.1)	13 (17)	
Overall, how much do sexual symptoms interfere with your everyday life? (*n*=525; 98%)				<0.001
Not at all	392 (75)	355 (80)	37 (45)	
A little	61 (12)	47 (11)	14 (17)	
Moderately	39 (7.4)	22 (5.0)	17 (20)	
Extremely	33 (6.3)	18 (4.1)	15 (18)	

Proportions may not add up to 100%, as they are rounded.
Unknown/missing values were not considered for the calculation of proportions, which may explain deviations from the sample of *n*=534 in the specific questions.
