# Supplementary material for: Exploring the intersection of functional recurrence, patient-reported sexual function, and treatment satisfaction after anterior buccal mucosal graft urethroplasty
Source: World J Urol. 2021 Mar 11;39(9):3533–9. doi: 10.1007/s00345-021-03648-y (PMC8510905; doi:10.1007/s00345-021-03648-y)
Supplement: Supplementary file 4 — Supplementary file4 (PDF 80 KB) [file 345_2021_3648_MOESM4_ESM.pdf]

Supplementary Table 3. Patient-reported sexual function after anterior 1-stage buccal mucosal graft urethroplasty, stratified by stricture location.

**Sexual Function Parameters**; *n* (%)	Overall (*n*=534)	Bulbar (*n*=438)	Penobulbar (*n*=57)	Penile (*n*=39)	*p* value
In case you have been able to get an erection, how would you rate the hardness of your erection? (*n*=531; 99%)					0.006
Sufficient rigidity	345 (65)	290 (67)	35 (61)	20 (51)	
Reduced rigidity	96 (18)	78 (18)	5 (8.8)	13 (33)	
No erection	90 (17)	67 (15)	17 (30)	6 (15)	
How was your postoperative erectile function compared to preoperatively, if rigidity was reduced or if there was no erection after surgery? (*n*=85; 46%)					0.097
Unchanged	59 (69)	40 (63)	13 (81)	6 (100)	
Worsened	26 (31)	23 (37)	3 (19)	0 (-)	
Does your glans fully swell during erection? (*n*=437; 82%)					0.6
Yes	401 (92)	334 (92)	36 (90)	31 (97)	
No	36 (8.2)	31 (8.5)	4 (10)	1 (3.1)	
How would you rate the amount or volume of semen when you ejaculate? (*n*=444; 83%)					0.3
As much as it always was	312 (70)	262 (71)	32 (78)	18 (56)	
Reduced volume	77 (17)	64 (17)	6 (15)	7 (22)	
No ejaculation	55 (12)	45 (12)	3 (7.3)	7 (22)	
How was your postoperative ejaculatory function compared to preoperatively, if semen volume was reduced or if there was no ejaculation after surgery? (*n*=43; 33%)					0.099
Unchanged	39 (91)	33 (92)	0 (-)	6 (100)	
Worsened	4 (9.3)	3 (8.3)	1 (100)	0 (-)	
In the last month, have you experienced any physical pain or discomfort when you ejaculated? (*n*=438; 82%)					0.8
No	402 (92)	337 (92)	37 (90)	28 (90)	
Yes	36 (8.2)	29 (7.9)	4 (9.8)	3 (9.7)	
Have you experienced scrotal or perineal numbness over the last couple of months? (*n*=528; 99%)					0.2
No	479 (91)	387 (90)	54 (95)	38 (97)	
Yes	49 (9.3)	45 (10)	3 (5.3)	1 (2.6)	
Has the angle of your erection changed after surgery? (*n*=512; 96%)					<0.001
No	472 (92)	394 (94)	50 (93)	28 (72)	
Yes	40 (7.8)	25 (6.0)	4 (7.4)	11 (28)	
Has the length of your penis changed since your surgery? (*n*=508; 95%)					0.011
No	456 (90)	379 (91)	48 (92)	29 (74)	
Yes	52 (10)	38 (9.1)	4 (7.7)	10 (26)	
Overall, how much do sexual symptoms interfere with your everyday life? (*n*=525; 98%)					0.075
Not at all	392 (75)	325 (76)	39 (70)	28 (72)	
A little	61 (12)	46 (11)	7 (13)	8 (21)	
Moderately	39 (7.4)	36 (8.4)	2 (3.6)	1 (2.6)	
Extremely	33 (6.3)	23 (5.4)	8 (14)	2 (5.1)	

Proportions may not add up to 100%, as they are rounded.
Unknown/missing values were not considered for the calculation of proportions, which may explain deviations from the sample of *n*=534 in the specific questions.
