# Supplementary material for: Exploring the intersection of functional recurrence, patient-reported sexual function, and treatment satisfaction after anterior buccal mucosal graft urethroplasty
Source: World J Urol. 2021 Mar 11;39(9):3533–9. doi: 10.1007/s00345-021-03648-y (PMC8510905; doi:10.1007/s00345-021-03648-y)
Supplement: Supplementary file 5 — Supplementary file5 (PDF 44 KB) [file 345_2021_3648_MOESM5_ESM.pdf]

Supplementary Table 4. Multifaceted characteristics of oral morbidity in 17 of 83 dissatisfied patients.

**Oral morbidity (*n*=17)**	*n* (%)
Intraoral pain	4 (24)
Intraoral numbness	11 (65)
Wound problems	2 (12)
Scar issues	5 (29)
Taste disorders	1 (5.9)
Slurred speech	1 (5.9)
Livid discoloration of the lip	1 (5.9)
Swollen lips	1 (5.9)

It is possible that a patient reported more than one of the above mentioned characteristics of oral morbidity.
